# Membranous Nephropathy (MN) Recurrence After Renal Transplantation

**DOI:** 10.3389/fimmu.2019.01326

**Published:** 2019-06-12

**Authors:** Patrizia Passerini, Silvia Malvica, Federica Tripodi, Roberta Cerutti, Piergiorgio Messa

**Affiliations:** ^1^Dialysis, and Renal Transplant Unit, Fondazione IRCCS Ca' Granda Ospedale Maggiore Policlinico, Milan, Italy; ^2^Department of Clinical Science and Community, Università degli Studi di Milano, Milan, Italy

**Keywords:** membranous nephropathy, recurrent membranous nephropathy, kidney transplant, proteinuria, anti-PLA2R antibodies, rituximab, prognosis

## Abstract

Primary membranous nephropathy (MN) is a frequent cause of NS in adults. In native kidneys the disease may progress to ESRD in the long term, in some 40–50% of untreated patients. The identification of the pathogenic role of anti-podocyte autoantibodies and the development of new therapeutic options has achieved an amelioration in the prognosis of this disease. MN may also develop in renal allograft as a recurrent or a *de novo* disease. Since the *de novo* MN may have some different pathogenetic and morphologic features compared to recurrent MN, in the present paper we will deal only with the recurrent disease. The true incidence of the recurrent form is difficult to assess. This is mainly due to the variable graft biopsy policies in kidney transplantation, among the different transplant centers. Anti-phospholipase A2 receptor (PLA2R) autoantibodies are detected in 70–80% of patients. The knowledge of anti-PLA2R status before transplant is useful in predicting the risk of recurrence. In addition, the serial survey of the anti-PLA2R titers is important to assess the rate of disease progression and the response to treatment. Currently, there are no established guidelines for prevention and treatment of recurrent MN. Symptomatic therapy may help to reduce the signs and symptoms related to the nephrotic syndrome. Anecdotal cases of response to cyclical therapy with steroids and cyclophosphamide have been published. Promising results have been reported with rituximab in both prophylaxis and treatment of recurrence. However, these results are based on observational data, and prospective controlled trials are still missing.

## Introduction

Membranous nephropathy (MN) is a podocytopathy histologically characterized by uniform thickening of the glomerular basement membrane due to the presence of sub-epithelial immune deposits. It can be secondary to a variety of causes, such as infections, autoimmune diseases, solid tumors, and medical reactions (1, 2) but in most cases (about 75%) it is an autoimmune kidney-specific disease defined as primary MN, that will be, together with its recurrence after kidney transplantation, the topic of this review.

Primary MN is one of the most common causes of nephrotic syndrome (NS) in adults and may lead to end-stage renal disease (ESRD) in the long term in some 40–50% of untreated patients (3–5), for whom kidney transplantation is the treatment of choice. However, after transplantation primary MN may develop again, either as a recurrence of the original disease or as a *de novo* form. When MN develops after renal transplantation, it negatively impacts on graft survival. Thus, the knowledge of the factors related with its recurrence and progression in the graft has important clinical implications.

Here we review the diagnostic approach, preventive measures and treatment strategies available for patients with recurrent MN.

## Primary MN

The natural history of primary MN follows three major clinical courses: spontaneous remission, persistent proteinuria and slow progression to ESRD; those patients with severe and un-remitting NS may also suffer from disabling and even life-threatening extra-renal complications, such as thrombo-embolic events and cardiovascular disease (6, 7).

Our understanding of the pathogenesis of primary MN has greatly improved in the past 10 years. In 2009, Beck et al. identified in the M-type phospholipase A2 receptor (PLA2R), a 185 kDa type I transmembrane glycoprotein expressed on glomerular podocytes, the major target antigens of the autoantibodies involved in primary MN. Circulating IgG4 anti-PLA2R antibodies are detectable in 70% of patients with active primary MN, and PLA2R staining colocalized with IgG4 in glomerular subepithelial deposits (8–13). A recent meta-analysis of 35 studies involving about 6,000 patients investigated the diagnostic test accuracy of serum anti-PLA2R antibodies and glomerular PLA2R antigen in discriminating between primary and non-primary MN. The overall sensitivity and specificity for serum anti-PLA2R antibodies was 65 and 97%, respectively and those for glomerular PLA2R antigen of 79 and 90%, respectively. The relatively low sensitivity of the serological test might suggest limitations in the presence of a negative result which should then require the need for a renal biopsy and for further research to establish potential secondary causes of MN. However, both serological and histological tests have high specificity, which means that a positive result indicates a highly likely diagnosis of primary MN. Besides a diagnostic role, in patients with primary MN, anti-PLA2R autoantibodies levels correlate with the immunological activity of the disease and provide information about its severity, long term outcome and treatment response (14, 15). This prognostic behavior of anti-PLA2R antibodies emphasizes their etiological role in primary MN, instead of simply being a biomarker of the disease (11, 16, 17).

In about 5% of cases of primary MN without anti-PLA2R, anti-thrombospondin type 1 domain-containing 7A (THSD7A) antibodies can be detected (18–20). In these patients a higher incidence of neoplasias has been reported, but the precise role of this antigen in this clinical context is currently a matter of investigation (21). No autoantibodies have yet been identified in the remaining primary MN patients (15–20%).

A genetic contribution to the development of primary MN is now well-established. A series of three independent genoma-wide association study in biopsy-proven cases of primary MN all of white ethnicity, showed that HLA-DQA1 and PLA2R1 haplotypes were associated with primary MN with high levels of statistical significance, with a combined odds ratio of about 80 for individuals homozygous for both risk alleles (22–25).

In patients with primary MN immunosuppression is reserved to those with severe unremitting NS, usually after at least 6 months of surveillance with symptomatic treatment (26). Traditional immunosuppressive regimens have included cyclical therapy with steroids alternated with an alkylating agent (cholambucil or cyclophosphamide) for 6 months, calcineurin inhibitors for 6 or more months, and more recently a B cell-targeted therapy with rituximab. Since patients with immunologically active disease can now be separated from those with inactive form, therapeutic initiatives can be tailored depending on the presence and levels of pathogenic antibodies, rather than empirically based on the clinical consequences of the glomerular immune damage such as proteinuria or reduced GFR. Serial changes in anti-PLA2R levels during treatment herald a response when falling titers indicate the immunologic remission of the disease.

## Recurrent MN

Like other autoimmune diseases, MN may recur after transplantation, leading to proteinuria and increasing risk of graft failure (27–31). The rate of recurrence may range between 30 and 45%, with higher rates reported by centres performing protocol biopsies (32, 33).

## Risk Factors for Recurrence

An early detection of patients who may develop recurrence would be helpful; unfortunately no baseline clinical characteristics have been reported to predict recurrence so far.

The genetic predisposition to the disease, due to the interaction of pathological HLA class II alleles with some PLA2R gene variants, has not been replicated in the post-transplant setting.

Some old studies suggested that MN would be more likely to recur in living than in deceased donor transplantations, assuming a relationship between genetic factors and MN susceptibility (34, 35). For example, Andrésdóttir et al. reported a cumulative rate of post-transplant disease recurrence of 70% in living related and of 21% in living unrelated donor transplants (36). In contrast, in the combined Lyon-Louvain Medical School series, recurrence was equally frequent in recipients of deceased and living donor transplant (37), and Dabade et al. described a higher number of recurrences among recipients of deceased donor kidneys (29). Referring to the European Renal Association-European Dialysis and Transplant Association (ERA-DTA) database, Pippias et al. showed by multivariate analysis that in transplanted patients with underlying primary MN, the 5- and 10-year death-adjusted graft survival was significantly better in living related donor transplants compared with deceased donors (38); their conclusions were against the reluctance to use living transplant donors in MN as well in other primary glomerulonephritis.

## Anti-PLA2R Antibodies in Recurrent MN

The identification of the main target antigen in primary MN, has focused the attention to the role played by aPL2R autoantibodies in the recurrence of the disease in kidney transplant.

There is now evidence that recurrent MN is triggered by the binding of recipient autoantibodies to the target antigen expressed on podocytes in the donor kidney. Stahl et al. first reported a case of recurrent MN with high titers of anti-PLA2R antibodies at the time of transplant and at recurrence (16). Since then, the relevance of monitoring anti-PLA2R activity to assess the risk of recurrence of primary MN has been extensively studied (see Table 1).

**Table 1:** PLA2R positivity and recurrent MN.

**References**	***N* (%) recurrence** **MN**	**PLA2R+ at Tx**	**PLA2R+ at recurrence**	**Median (range)** **pre-Tx titer in rMN**	**PPV/NPV** **(%)**	**COMMENT**
Kattah et al. (39)	18/26 (69)	10/17 with recurrence 2/7 without recurrence	7/18 in post-Tx period	19 (0–1,200) Western blot	83/42 for pre-Tx PLA2R	
Quintana et al. (40)	7/21 (33)	6/7 with recurrence 5/14 without recurrence	6/7	741 (11-1500) ELISA	85/92	-Recurrence significantly associated with high level PLA2R+ before Tx (*P* = 0.03) -A cut–off level of 45 U/mL during pre-Tx period predicted rMN with a sensitivity of 85%, specificity of 85% and a NPV of 92%
Gupta et al. (41)	6/16 (37)	5/6 with recurrence 0/10 without recurrence	5/6	82 (31-1500) ELISA	100/91	Combining data with those of Quintana et al., Pre-Tx APLA2R >29 RU/mL predicted rMN with a sensitivity of 85% and a specificity of 92%
Debiec et al. (11)	10/10 (100)	4/4 with available serum	5/10	NA	NA	
Seitz-Polski et al. (17)	5/13 (38)	4/5 with recurrence 6/8 without recurred]nce	4/5	748 (137-3000)	40/80	Presence of PLA2R at the time of Tx does not imply recurrence (*P* = 0.6). Positive PLA2R activity during follow up(>6 months) significantly correlates to recurrence (*P* = 0.048)

*PLA2R, anti-phospholipase A2 receptor; Tx, transplant; PPV, positive predictive value; NPV, negative predictive value; NA, not available*.

The percentage of transplant candidates with primary MN who have circulating anti-PLA2R antibodies is the same as in the general primary MN population (70–80%) (39, 42, 43). These patients have a 60–76% risk of hystological recurrence, vs. a significantly lower risk in antibody negative patients (28–30%) (17, 39, 40, 42). Kattah et al. reported that among 26 transplanted patients with underlying primary MN, 18 developed recurrent MN and 8 did not. In the recurrent group, 10 out of 17 were anti-PLA2R positive at the time of transplantation (55%), as well as 2/7 in the non-recurrent group (25%). In their cohort, the positive predictive value (PPV) of pretransplant anti-PLA2R antibodies for recurrent MN was 83% and the negative predictive value (NPV) was 42%, thus, in their study, 58% of seronegative patients at transplantation may also have recurrence (39). Quintana et al. studied 21 Spanish patients with primary MN before transplantation. The recurrence of the disease was significantly correlated with a positive ELISA assay at graft biopsy (*P* = 0.017) or anti-PLA2R seropositivity before transplantation (*P* = 0.03). In order to better define the pretransplant anti-PLA2R cut-off level that could more accurately predict rMN, the Authors performed a receiver operating characteristic analysis (ROC), and found that an anti-PLA2R cut-off of 45 RU/mL pretransplantation, was predictive of primary MN recurrence with a sensitivity of 85.3%, a specificity of 85.1%, a NPV of 92% and an area under the curve of 90.8 % (*P* = 0.03) (40). Likewise, Gupta et al. showed that 5 out of 6 transplanted patients (83%) with a biopsy-proven diagnosis of recurrent MN, were anti-PLA2R positive pretransplant, with a median titer of 82 RU/mL vs. none of the 10 without anti-PLA2R before surgery. Based upon their results, they found that an ELISA titer above 30 RU/mL, provided a sensitivity of 83%, a specificity of 100%, a PPV of 100% and a NPV of 91% for recurrence. Pooling together these data with those from Quintana et al. (*n* = 21), their ROC analysis showed that a pretransplant anti-PLA2R cut-off titer above 29 RU/mL was predictive of recurrence with a sensitivity of 85% and a specificity of 92% (41). These *quantitative* results, which have never been reported previously in a clinical cohort, may contribute to a better identification of patients at risk of recurrence for prognostic and therapeutic purposes.

The strong correlation between anti-PLA2R titer and the risk of primary MN recurrence stressed by these studies was not confirmed by others, which reported a significantly lower PPV in seropositive patients (40 to 50%). A study by Debiec et al. reported that anti-PLA2R antibodies were implicated in only 5 of 10 patients with recurrent MN; moreover, by studying the levels of anti-PLA2R and the timing of the recurrence of the disease, they showed a marked heterogeneity in the kinetics and titers of anti-PLA2R, showing no simple association between anti-PLA2R activity in transplantation and the risk of recurrence (11). Similarly, Seitz-Polski et al. found that only a positive IgG4 anti-PLA2R1 activity during the follow-up (more than month 6) was significantly related to the recurrence (*P* = 0.0048): among 10 anti-PLA2R seropositive patients at transplantation, recurrence occurred only in the four patients who maintained seropositivity during their follow up (17).

Several biases in these studies may explain the opposing results reported without lessening the predictive value of anti-PLA2R for recurrence risk. The main one concerns the wide variability of the therapeutical protocols applied. A more intensive induction immunosuppression and the modulation of the maintenance immunosuppressive therapy could interfere with the autoantibody production and their circulating post-transplant levels (39). Consequently recurrence may be anticipated or delayed. This offers an explanation for the contrasting results reported by Quintana et al. and Seitz-Polski et al. who treated with the induction therapy 42 and 60% of their patients, respectively. Another bias could be represented by the lack of quantification of anti-PLA2R titer in some studies. This may overestimate a very low titer, not reaching the cut-off level predicting the recurrence. Finally, the use of renin-angiotensin system blockers associated with calcineurin inhibitors could have masked, in some studies, a subclinical recurrence with low grade proteinuria.

In summary, the available studies suggest that the benefit of anti-PLA2R antibodies titration lies in identification of recipients at risk of recurrence and possibly achieving an early diagnosis of recurrent MN. However, anti-PLA2R remains an imperfect diagnostic tool because seropositivity does not necessarily imply recurrence, as well as seronegativity does not necessarily exclude it.

## Outcome of Recurrent MN

Two peaks of MN recurrence have been described: an “early recurrence” within the first 6–12 months after transplantation, and a late-onset recurrence at about 5 years after transplant (29, 42).

Early recurrence, most likely due to deposition of circulating anti-PLA2R present at transplantation in the graft (32), is usually detected by protocol biopsies in patients with absent or mild clinical manifestation of the disease (28, 29). The histologic features of early recurrence, that can be observed as early as 1–2 weeks after surgery (40), are often different from those observed in patients with clinically evident disease. For example, light microscopy may not identify subepithelial deposits and basement membrane spikes, and C3 staining appears to be weak or absent at immunofluorescence (29). In contrast, at electron microscopy extensive podocyte foot process fusions and subepithelial electron dense deposits are seen. A fundamental finding for diagnosis is the presence of PLA2R staining within the glomerular immunodeposits (11, 44).

Late MN recurrence is clearly the result of new post-transplant production of anti-PLA2R antibodies. It is usually diagnosed in patients with a progressive increase in proteinuria evolving in a full-blown NS. Histologically, the features of late recurrence resemble those observed in the native kidney, and the glomerular lesions can be associated with vascular and tubulo-interstitial damage caused by rejection, CNI toxicity or infection.

It is clear that a protocol biopsy provides the earliest diagnosis of recurrent MN. At this point, it would be essential to verify whether or not a subclinical recurrence, that is usually present when the protocol biopsy is performed, may have consequences on the graft prognosis. If so, the protocol biopsy may be essential to obtain a diagnosis before the progression of the disease to a more severe stage. Several studies have shown that a clinically silent disease at the time of biopsy typically progresses to overt proteinuria and NS when followed over time (29, 32). A study by El-Zoghby et al. reported an increasing level of proteinuria from 331 mg/d to 1409 mg/d during a 19 months follow up in 14 patients with recurrent MN. The authors also found that the earlier diagnosis obtained in patients diagnosed by protocol biopsies was associated with a significantly lower proteinuria compared with patients in whom the graft biopsy was performed on clinical indications (28). In addition, Grupper et al. reported a persistent histological activity in both untreated patients without progressive proteinuria, and in rituximab treated patients who obtained a complete or partial remission (42). These results confirmed that recurrent MN may be considered a progressive disease, with a high risk of progression even in the presence of mild proteinuria (30, 32, 45). This progressive outcome should not be surprising since MN in a transplant candidate is indicative of a disease which has led to ESRD.

The behavior of anti-PLA2R antibodies post transplantation may be variable. In patients with recurrent MN, serial titration of autoantibodies is relevant to assess the risk of disease progression and the chances of response to treatment, and may help to differentiate diagnosis in recipients with a history of primary MN who develop proteinuria. The persistence or reappearance of anti-PLA2R after transplantation may herald a more aggressive disease with a lower response to immunosuppressive therapy and a longer time to remission. In patients who obtain immunological and clinical remission after treatment, reappearance of anti-PLA2R antibodies may herald the relapse of the disease (39). However, disappearance of anti-PLA2R shortly after transplant, due to immunosuppression or unknown factors related to antigen exposure in the allograft, is not always reassuring. In some cases this finding corresponds to a lower risk of recurrence but in others the few circulating autoantibodies can be trapped in the graft and although undetectable in the serum may accumulate in the glomeruli and cause recurrence despite apparent immunological remission (11, 39, 46).

It must be kept in mind that in seronegative patients with recurrent MN, it is possible that other autoantibodies to podocyte antigens, like THSD7A or the cytoplasmic antigens, could be involved, but their role in recurrent MN is still undefined (18).

The impact of recurrence on renal survival has been evaluated differently. Some investigators reported no significant difference in patient and graft survival between patients with and without recurrence (32), while others have shown a progression to ESRD between 60 and 65% of patients with rMN, after a mean interval of 4 years from diagnosis (37, 47). Data from the largest series to date support the negative influence of recurrent disease on allograft survival. Using the ERA-EDTA registry database, Pippias et al. assessed death-adjusted graft survival in 708 patients with primary MN compared with those in 7,181 patients with autosomal dominant polycystic kidney disease, in which the native kidney disease cannot recur. The 5-, 10-, and 15-year adjusted relative risk of death-adjusted graft failure was, respectively of 1.46 (95% CI: 1.18–1.82), 1.60 (1.34–1.91), and 1.65 (1.40–1.95). These negative effects were ascribed to detrimental effects of MN recurrence (38).

In summary the results of these studies suggest that recurrent MN has a more insidious course than primary MN, with a potential histologic progression regardless of the clinical status that can lead to unexpected negative consequences on graft function. Surveillance of recurrence in the kidney graft recipients with biopsy may provide an opportunity to prevent further allograft dysfunction at an early stage, when the disease may theoretically be more responsive to therapy (48).

## Treatment of Recurrent MN

Symptomatic treatment of recurrent MN with diuretics, ACE-inhibitors, ARB, hypolipaemic drugs, and anticoagulants, may help to attenuate the signs and symptoms related to the NS. There is evidence however, that these measures fail to prevent the progression of the disease in the majority of patients (29).

Currently, there are not established guidelines supporting therapeutical strategies to prevent and treat recurrent MN. Steroids, calcineurin inhibitors and antiproliferative agents, which have been used in patients with primary MN do not have a protective effect in the prevention of recurrence, and there is no suggestion that modifying traditional post-transplant immunosuppression may reduce its risk. Sometimes the post-transplant induction immunosuppression may obtain a temporary inhibition of anti-PLA2R production but seronegativity does not exclude deposition of PLA2R in the glomeruli. The traditional regimens for primary MN are of little or no benefit in recurrent MN. Calcineurin inhibitors that may be effective in primary MN (49, 50) do not seem to improve the clinical course of recurrent MN (29, 37, 47, 51). Anecdotal cases of response to cyclical therapy with steroids and cyclophosphamide were reported (27, 52) but cytotoxic agents may result in potentially dangerous overimmunosuppression in transplanted patients.

With a better understanding of the pathogenesis of primary MN, agents acting against B cells constitute a more selective form of therapy. Rituximab has emerged as a promising therapeutic option in patients with primary MN in the native kidney (4, 53–55). It binds specifically to CD20, an antigen expressed by most human B lymphocytes, resulting in rapid and sustained depletion of circulating and tissue B cells (56). The first report of successful rituximab treatment in primary MN appeared in 2002. Since then, several Authors have published their positive experience, although still randomized controlled studies are not available. Overall, complete remission (CR) occurs in about 15–20% and partial remission (PR) in 35–40% of patients. The clinical response to rituximab has been described in relation to its effects on anti-PLA2R titers. Since changes in anti-PLA2R levels (immunologic remission) precedes proteinuria reduction (clinical remission) by months, monitoring anti-PLA2R titer may help to earlier understand the treatment effectiveness as compared with the clinical response of proteinuria.

Good results with this agent are also reported in recurrent MN. Gallon et al. first described a case of successful treatment of recurrent MN with 4 weekly doses of rituximab 375 mg/m^2^, which obtained a significant reduction in proteinuria from 16 g/day to 0.5 g/day at 3 years (57). Subsequently, several other authors have published on the beneficial effects of rituximab in this setting on case reports or small series (see Table 2). Three larger series are available in the literature. A study by El-Zoghby et al. reported on 8 patients with recurrent MN and a mean proteinuria of 4.4 g/24 h, treated with 2 doses of rituximab, 1,000 mg iv each, given 2 weeks apart. After rituximab there was a prompt and prolonged reduction in CD19 count in all patients. The count remained below the normal range in the 7 patients tested at 12 months, and in the 4 patients tested at 24 months. Treatment obtained a progressive increase in the percent of responses over the first 24 months following rituximab, when a CR or PR was present in 6 out of 7 patients. Among responders, one patient relapsed and was successfully retreated. Two patients developed serious infections within the year following treatment (1 pneumonia, 1 histoplasmosis). Post treatment biopsies showed resolution of electron dense immune deposits in 6/7 cases (28). Gupta et al. treated 6 patients with recurrent MN with 1-2 doses of rituximab (375 mg/m^2^ two weeks apart). In all patients proteinuria improved from a median of 6 g/d (range 3.6–19.6) to 0.6 g/d (range 0.1–2.2) after a median follow up of 5.8 years. Among the 5 patients with positive anti-PLA2R pretransplant and at the time of recurrence, titers fell to a median of 4 RU/mL (41). A recent study from the Mayo Clinic reported the response to rituximab in a larger series of 17 patients with recurrent MN and progressive proteinuria, who received rituximab, 2 doses of 1,000 mg each given intravenously 2 weeks apart. After therapy, 9/17 patients achieved CR (53%), 5 PR (29%), and 3 (18%) had no apparent response to therapy. Of the 9 patients who achieved CR after treatment, all maintained remission for 20–92 months and did not require additional therapy. Among the 5 patients with PR, 2 required retreatment with rituximab after more than 24 months for increasing proteinuria and both of them achieved the PR again. Within 2 years after anti-CD20 therapy, 5 out of 17 patients (29%) developed serious infections requiring hospitalization (3 urosepsis, 1 histoplasmosis, and 1 pneumonia). No patient died. Post treatment biopsies were obtained in 15/17 patients. In 6 of them (40%), there was a resolution of the histologic findings (5 with CR and 1 with PR), but in 9 out of 15, immunofluorescence and electron microscopy showed persistence of active MN (4 with CR, 3 with PR and 2 non-responders) (42).

**Table 2:** Results of treatment of recurrent MN with rituximab.

**References**	**Dose of rituximab**	**Number of treated patients**	**Percent CR/PR**	**Proteinuria g/day**
Gallon et al. (57)	4 weekly doses (375 mg/m^2^)	1	NA	From 16 to 0.5
El-Zoghby et al. (28)	2 doses (1,000 mg) 2-week apart	8	55/30	NA
Sprangerset al. (32)	4 weekly doses (375 mg/m^2^) or 2 doses (1,000 mg) 2-week apart	4	NA	From 4 to 1.8
Debiec et al. (58)	2 doses (375 mg/m^2^) 2-week apart	1	NA	From 5.1 to 0.4
Spinner et al. (59)	Single dose 100–1,000 mg (median 200)	20	40/15	NA
Gupta et al. (41)	1–2 doses 375 mg/m^2^ 2-week apart	6	NA	From 6 to 0.6
Grupper et al. (42)	2 doses (1,000 mg) 2-week apart	17	53/29	NA

*CR, complete remission; PR, partial remission*.

Currently, there are not data on preemtive use of rituximab.

Rituximab is generally considered effective, and more tolerated as compared to alkylating agents. Another advantage with rituximab is the unnecessary modification of the anti-rejection therapy. However, treatment may complicate with infections in transplanted patients receiving 2–3 additional immunosuppressants. Therefore, it has been suggested that in recipients with higher risk of infections such as older recipients, the dose of anti-CD20 may be reduced by half (48, 60). Treatment rapidly decreases anti-PLA2R. B-cell depletion lasts longer in transplanted patients than in native MN (28), but it is usually necessary to wait more than one year to achieve clinical remission (28, 55, 61). The rate of response to treatment resulted to be higher in recurrent MN than in native MN. This may be due to the earlier diagnosis and treatment of the disease in many patients, and to the additional beneficial effects of the post-transplant immunosuppression (28). Serological monitoring for anti-PLA2R levels may represent the earlier biomarker of immunological response. The change in anti-PLA2R titer precedes the decline of proteinuria. Therefore, serial titration of the antibody can be used to predict response to therapy as well as the possibility of relapse (28).

The optimal dose of rituximab for recurrent MN is still unknown. Usual dose of rituximab in native MN consists of 375 mg/m^2^ intravenously once weekly for 4 weeks or the alternative protocol of 2 doses of 1 g each intravenously 2 weeks apart. The regimen can be repeated after 6 months if B cells are above 15/μL or elevated anti-PLA2R levels persist. So far the literature has not provided a clear indication on the most effective regimen. It can only be said that, unlike primary MN in the native kidney, the progressive course of recurrent MN requires treatment at the earliest stage rather than an initial period of observation. Infact, in primary MN the amount of proteinuria relates to prognosis (49) and spontaneous remission of NS may occur in about 1/3 of patients (62). In contrast, recurrent MN appears to have a much higher chance of progressing even when only minimal proteinuria is present, and a high rate of success is reported starting treatment when proteinuria is approaching one gram per day (29). These observations justify an early treatment of recurrent MN in allografts.

In summary, although treatment of recurrent MN is still far from satisfactory, some measures seem to be helpful. Rituximab is currently considered the first line therapy but prospective trials in this setting are needed to confirm the available data. In patients heavily immunosuppressed caution is recommended in order to avoid severe and even life-threatening infections. The ideal dose is another important aspect to be evaluated.

Among transplanted patients who do not respond to rituximab, cytotoxic agents such as cyclophosphamide may be cautiously used. If such a therapy is considered, antimetabolic agents such as mycophenolate mofetil or azathioprine should be discontinued and patients should be followed up closely for bone marrow suppression, infection, and malignancy. There are no rigorous studies that have examined the effects of cytotoxic agents in recurrent MN. Finally, a successful use of a proteasome inhibitor (bortezomib) has been reported in recurrent MN with persistent proteinuria refractory to rituximab (63).

## Conclusion

Due to the overall longer survival of transplanted kidneys, recurrency of native diseases has become an increasing cause of allograft failure (33). In recurrent MN, persistent NS can cause 10% of graft loss within 4.2 ± 2.7 years after transplantation (28). There is evidence in the literature that an approach focused on early diagnosis and prompt therapy can improve graft survival. In this setting the allograft biopsy and the titration of anti-PLA2R autoantibodies are the main diagnostic and prognostic tools. Evaluation of anti-PLA2R antibodies before surgery may predict the risk of recurrence of primary MN: in patients with persistently high anti-PLA2R levels preemptive treatment with anti-CD20 could hypothetically be effective in preventing recurrence. However, data on this subject are lacking.

**Figure 1:**
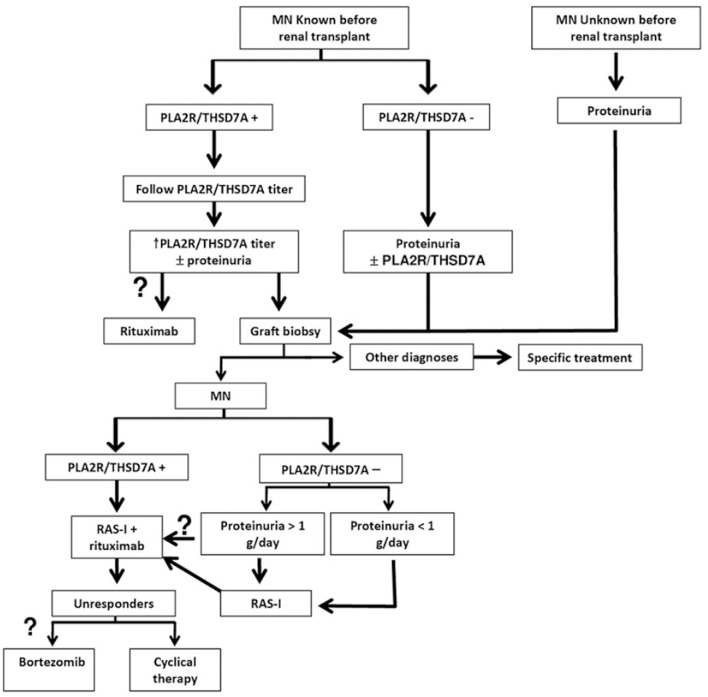
Antibody-guided diagnosis and treatment algorithm for recurrent MN. RAS-I Renin- angiotensin system inhibitors, PLA2R anti-phospholipase A2 receptor, THSD7A anti- thrombospondin 1 type 1 domain containing 7A. Question marks on optional points.

The protocol biopsy remains the gold standard for earlier diagnosis of recurrent MN, prior to its clinical presentation, at a time point where specific intervention may be more effective. In transplanted patients with underlying MN, the subsequent evaluation of anti-PLA2R antibodies level provides information about the immunological activity and outcome of the disease and enables clinicians to adjust the immunosuppressive therapy to the patient's needs. Thus, in biopsy proven recurrent MN, the immunological activity (anti-PLA2R levels) and the clinical activity (proteinuria degree) of the disease should be followed closely. Regardless of PLA2R levels in patients with proteinuria reaching 1 g/day, therapy should be considered. Data regarding rituximab treatment, although still limited, predict favorable results in the treatment of recurrent MN. Prospective randomized trials are needed to confirm the reported results. In case of poor response or intolerance to rituximab, the cyclical therapy with steroids and cytotoxic agents or with bortezomib may be attempted (see Figure 1). Serial measurement of antibody levels during treatment may be useful to predict responsiveness to therapy and thereafter may be useful to predict relapse. We report our clinical practice algorithm in figure 1 (see Figure 1).

## Author Contributions

PP, SM, FT, RC, and PM conceived the article contents, prepared the manuscript, and endorsed the final draft submitted.

### Conflict of Interest Statement

The authors declare that the research was conducted in the absence of any commercial or financial relationships that could be construed as a potential conflict of interest.
